# High confidence, yet poor knowledge of infant feeding recommendations among adults in Nova Scotia, Canada

**DOI:** 10.1111/mcn.12903

**Published:** 2019-11-27

**Authors:** Kathleen Chan, Kyly C. Whitfield

**Affiliations:** ^1^ Department of Applied Human Nutrition Mount Saint Vincent University Halifax Nova Scotia Canada

**Keywords:** complementary feeding, breastfeeding, health promotion, public health, infant feeding decisions, knowledge

## Abstract

In Canada, adherence to the national ‘Nutrition for Healthy Term Infants' recommendations of infant and young child feeding (IYCF; 0‐24 months) is suboptimal. While maternal knowledge of IYCF is commonly assessed, that of the general public has rarely been explored. Our objective was to assess the knowledge of, and confidence in answers to, Canadian IYCF recommendations among a diverse sample of adults in Nova Scotia, Canada. Between March and May 2018, a self‐administered questionnaire examining IYCF knowledge, self‐rated confidence, and sociodemographic information was conducted among Nova Scotians (≥19 years) in public locations. We surveyed 229 adults; 60% (n=134) were women. Mean (95% CI) age was 44 (41,46) years, 73% self‐identified as white, 77% were born in Canada, and 69% were parents. Knowledge deficits were: age to terminate breastfeeding (18.3 (16.7,19.9) months; recommendation: ≥24 months), age to introduce solids (9.2 (8.2,10.2) months; recommendation: 6 months), vitamin D supplementation (10% correct), and optimal complementary foods (only 37% indicated iron‐rich foods). Correct IYCF knowledge was lower among men, non‐parents, young adults (19‐29 years) and low‐income adults (<$50,000/year). Mean self‐rated confidence (out of 10) was high (7.2 (6.9,7.5)), and not different (p>0.05) between correct and incorrect responses for: best food for a newborn, age to terminate any breastfeeding, and age to start family meal foods. We found low knowledge of IYCF guidelines, yet high confidence in responses regardless of accuracy, among adults in Nova Scotia. General public knowledge deficits may contribute to an unsupportive culture around IYCF practices and low adherence to current recommendations.

**Key Messages**

- Despite knowledge that sociocultural norms heavily influence infant feeding practices, to date most research investigating knowledge of infant and young child feeding recommendations focusses on caregivers and healthcare practitioners, not the general public.
- Among our diverse sample of adult Nova Scotians, we identified knowledge deficits with regards to the age to start complementary feeding (responses averaged 9 months, rather than the recommended 6 months), and what the optimal first foods were (only one third of participants identified recommended iron‐rich foods). Only one tenth of respondents correctly noted that vitamin D supplementation was required by exclusively breastfed infants in Canada.
- Both global and Canadian guidelines recommend continued breastfeeding for 2 years and beyond, but the mean age to terminate any breastfeeding proposed by our study participants was 18 months, suggesting a discomfort with older infants and young children breastfeeding.
- We found that infant and young child feeding knowledge was lowest among men, non‐parents, young adults (19‐29 years) and low‐income adults (<$50,000/year). Infant and young child feeding knowledge did not differ by education status.
- Self‐rated confidence in responses to infant and young child feeding knowledge questions was high among all population sub‐groups, regardless of the accuracy of responses.

## 1 INTRODUCTION

Improving infant and young child feeding (IYCF) practices for optimal maternal and infant health is an ongoing global health priority (World Health Organization, 2014). Recommended IYCF practices such as exclusive breastfeeding to 6 months with continued breastfeeding to 2 years and beyond, and the timely introduction of appropriate complementary foods, are promoted globally for their health benefits for both mother and child (Fewtrell et al., 2017; Kramer & Kakuma, 2012; Smith & Becker, 2016; Victora et al., 2016). In Canada, Health Canada, the Canadian Paediatric Society, Dietitians of Canada, and the Breastfeeding Committee for Canada work collaboratively to publish two sets of national IYCF recommendations, titled Nutrition for Healthy Term Infants (NHTI) from birth to six months (Critch, 2013), and NHTI from six months to 24 months (Critch, 2014).

Despite efforts to promote NHTI recommendations in Canada, rates of adherence remain moderately low. For instance, in 2018 Statistics Canada surveyed women who had given birth within the previous 5 years and found that 92% of Canadian mothers initiated breastfeeding, however only 37% breastfed exclusively to 6 months (Statistics Canada, 2018). In Nova Scotia, adherence was even lower than the national average, with 88% and 22% initiating breastfeeding and breastfeeding exclusively to 6 months, respectively (Statistics Canada, 2018). Adherence to NHTI guidelines is even lower when the full range of IYCF recommendations for breastfeeding and complementary feeding are considered as a whole. A recent longitudinal study in Ontario, Canada followed 325 mothers with healthy infants from birth to 6 months, and reported that only 2% of participants followed all of the NHTI recommendations (Fegan, Bassett, Peng, & Steel O'Connor, 2016). This is not surprising given the low exclusive breastfeeding rates in Canada, however, when breastfeeding exclusivity was removed, this figure only rose to 5% adherence to all other recommendations (Fegan et al., 2016).

Sociocultural factors such as public beliefs and cultural norms are key determinants of breastfeeding initiation and duration, and complementary feeding practices (Li, Fridinger, & Grummer‐Strawn, 2002; Rollins et al., 2016). An unsupportive culture around breastfeeding has been identified as a key barrier to improving breastfeeding outcomes both in Nova Scotia and globally (Kirk, Sim, Hemmens, & Price, 2012; Rollins et al., 2016; Russell & Ali, 2017). Infant feeding decisions are made within a social context; partners and grandparents can have a significant impact on breastfeeding outcomes (Mueffelmann, Racine, Warren‐Findlow, & Coffman, 2015; Vaaler et al., 2011), infant feeding decisions are often made before becoming pregnant (Rempel & Rempel, 2004), and public support can affect wider policy decisions (Kirk et al., 2012; Li et al., 2004). Assessing cultural norms and beliefs around infant feeding in the general public may provide insight into broader IYCF outcomes.

Attitudes towards breastfeeding are positively associated with breastfeeding knowledge among various populations (Chan & Whitfield, 2019). Despite the strong influence cultural and societal norms are known to play in dictating IYCF behaviours, the majority of research to date has focused on quantifying the IYCF knowledge of caregivers (parents, parents‐to‐be, and healthcare providers), and has focused mainly on breastfeeding (Chezem, Friesen, & Boettcher, 2003; Fairbrother & Stanger‐Ross, 2010; Pound, Williams, Grenon, Aglipay, & Plint, 2014). The limited research exploring knowledge and attitudes toward complementary feeding has been largely limited to caregivers in low‐ and middle‐income countries, and is not applicable to a Canadian population (Hackett, Mukta, Jalal, & Sellen, 2015; Katepa‐Bwalya et al., 2015; Mohammed, Ghazawy, & Hassan, 2014). Previous research has assessed IYCF knowledge and self‐rated confidence among caregivers to identify knowledge deficits and provide insight on practices (Brodribb, Fallon, Jackson, & Hegney, 2008; Pound et al., 2014), however the potential disconnect between the general population's IYCF beliefs and evidence‐based IYCF practice recommendations has not been explored. As such, the overall aim of this study was to assess the knowledge of, and confidence in knowledge of, Canadian IYCF recommendations among a diverse sample of adults in Nova Scotia.

## 2 METHODS

### 2.1 Design

This exploratory, cross‐sectional survey used an open‐ended, self‐administered paper questionnaire to assess knowledge of Canadian IYCF recommendations, the NHTI recommendations, and self‐rated confidence in responses to these knowledge questions. Purposive and convenience sampling were employed to reach a wide population, and open‐ended questions were used to better understand current trends in IYCF knowledge in the province. This study was approved by the Mount Saint Vincent University Research Ethics Board (MSVU UREB 2017‐068).

### 2.2 Setting and recruitment

Data was collected from 11 purposively selected sites across Nova Scotia to promote sample diversity in participant age, household income, education level, immigration status, parental status, as well as urban and rural residency. The seven urban sites in the Halifax Regional Municipality included a community centre, church, toddler play group, preschool, recreation/fitness centre, long term care facility, and university. Two sites were selected in rural Eastern Nova Scotia (a community centre in Big Bras D'Or and grocery store in Sydney, Cape Breton) and two in rural Western Nova Scotia (two grocery stores, in Barrington Passage and Yarmouth). All data were collected between March and May 2018.

Eligibility criteria included adults ≥19 years of age, who were proficient in basic English, and living in Nova Scotia at the time of the study. Participants were recruited via convenience sampling (booth with poster) at each purposively‐selected site, completed the questionnaire on location (10‐15 minutes), and were remunerated with a gift card for their participation.

### 2.3 Measurement of IYCF knowledge

Several questionnaires have been developed to measure infant feeding knowledge and attitudes, such as the Infant Feeding Test Form‐A (Grossman, Harter, & Hasbrouck, 1990), the Breastfeeding Behavior Questionnaire (BBQ) (Libbus, 1992) and the Iowa Infant Feeding Attitudes Scale (De La Mora, Russell, Dungy, Losch, & Dusdieker, 1999). However, these questionnaires focus solely on breastfeeding, were designed for caregivers (i.e. to predict behaviour), use only close‐ended questions (Casal, Lei, Young, & Tuthill, 2017), and are not specific to the full range of Canadian NHTI recommendations. As such, a novel questionnaire was developed for this study. IYCF knowledge was assessed by asking questions from each guideline set forth in the NHTI; see **Box** 1. Participants were then asked to self‐rate their confidence in the accuracy of the answers they provided for each of the knowledge questions using a visual analog scale (VAS; out of 10, from ‘not at all confident' to ‘very confident'). VAS scores are widely used in various fields to measure subjective experiences, such as pain (Boonstra, Schiphorst Preuper, Reneman, Posthumus, & Stewart, 2008), sensory evaluations (Ramaekers et al., 2014), well‐being (Johnson, Culverwell, Hulbert, Robertson, & Camic, 2017), and confidence in knowledge and skills (Finch et al., 2013). VAS has been preferred over ordinal Likert scales as they are often more responsive, and easy to use and understand, particularly among a diverse population (Hasson & Arnetz, 2005). This study is the first to assess self‐rated confidence in IYCF knowledge among the general public.

**Box 1:** Infant and young child feeding knowledge questions posed in our questionnaire, with corresponding Nutrition for Healthy Term Infants (NHTI) recommendations.

Question	NHTI recommendation
1. What do you think is the best food for a new baby?	Breastmilk
2. If a mom is breastfeeding, how long do you think she should exclusively breastfeed (only give breast milk to a baby)?†	6 months
3. If a mom is exclusively breastfeeding, is there anything else she needs to give her baby (eg. food, drink, or supplement)?	Daily vitamin D supplement (10μg) *Medication if needed*
4. Are there any reasons that you know of not to breastfeed? a) For mom? b) For baby?	Maternal reasons to not breastfeed include rare illnesses as well as certain medication use or treatments.‡ Infant reasons include rare intolerance to breastmilk (such as galactosemia).
5. At what age do you think a baby or young child should stop breastfeeding?	24 months or beyond
6. At what age do you think a baby should start regularly eating solid foods such as purees, in addition to breastfeeding?	6 months
7. a) What would you consider the best first solid food for a baby? b) Why is this the best first solid food for a baby?‡	Iron‐rich foods (meat and alternatives, iron fortified cereal), because breastmilk alone no longer meets the iron requirements of an infant after 6 months.
8. At what age do you think babies should start eating lumpy textures?	Minimum 9 months
9. At what age do you think babies or young children should start eating from the foods available in a regular family meal (may be modified for texture)?	12 months
10. Do you think babies or young children should drink cow's milk?§ a) At what age should a baby or young child first drink cow's milk? b) What kind of cow's milk should they drink?	Cow's milk is not necessary for infants and young children. If introduced, 3.25% milk fat should be consumed, and delayed until between 9‐12 months of age.
11. At what age do you think a baby or young child should start drinking from an open cup (not bottle or sippy cup)?‡	Open cups can be introduced as soon as other fluids are introduced (i.e. ≥6 months)
12. Do you know of any solid foods that a baby or young child under 2 years should not eat?	• Choking hazards (hard, small, round or smooth and sticky solids) • Raw or undercooked meat, poultry or fish • Raw or lightly cooked eggs • Unpasteurized milk and milk products or juices • Honey¶ (<1 year)

† Maternal illnesses cited in NHTI include: HIV, herpes lesions on both breasts, untreated infectious tuberculosis, any severe illness that prevents the mother from caring for her infant, and certain drugs or treatments (however most common prescriptions are acceptable).

‡ Participant responses were too general to compare to NHTI recommendations, therefore were not analyzed for correctness in Table 3.

§ Responses to question 10 were not analyzed for correctness, because cow's milk is not explicitly recommended for consumption, however 10. a) and b) are included in analysis.

¶ Honey was accepted as a contraindicated food before 2 years, because the question did not specify age ranges below 2 years.

Demographic information (age, marital status, gender, household income, racial or cultural group identity, education, employment) as well as information on potential exposure to IYCF recommendations (parental status, and breastfeeding experience) was also collected.

### 2.4 Data Collection

Teams of two research assistants recruited participants during predetermined times after receiving approval to set up a booth at the data collection sites. Research assistants approached potential participants, and screened interested participants for inclusion criteria. Of the 259 people invited to participate, a total of 229 participants completed the questionnaire (n=27 were not eligible, n=3 did not complete the questionnaire). Participation was anonymous and voluntary, and written, informed consent was obtained from all participants.

### 2.5 Data Analysis

Data analysis was performed using SPSS for Windows v.24 (IBM Corp.) with a significance level of *p*<0.05. Descriptive statistics were computed and shown as *n* (%) or mean (95% CI) for sociodemographic data, answers to IYCF knowledge questions, comparisons to the NHTI principles (correct/incorrect responses), and self‐rated confidence in responses to the IYCF knowledge questions. Responses to the IYCF knowledge questions were coded as correct or incorrect, as determined by NHTI principles, and then analyzed for all participants and by the following sociodemographic variables: gender, age group, household income, parent status, and education level. Some questions were not assessed for correctness due to the nature of the question or responses; for example, since cow's milk consumption is not explicitly recommended, only the age of introduction and type of milk was assessed for accuracy. Answers related to age to terminate exclusive breastfeeding, contraindications to breastfeeding, explanations for a best first solid food, and age to drink from an open cup were too general to compare to the NHTI principles and were therefore excluded from analysis.

χ^2^ tests were used to assess differences in sociodemographic characteristics by gender, and in correctness of IYCF knowledge responses by gender, age group, household income, parental status, and education level. Bonferroni post‐hoc analysis was used to assess differences in correctness of IYCF knowledge responses by age group and household income category. VAS confidence scores were not normally distributed (Shapiro Wilks test p<0.05), and could not be transformed to a normal distribution, therefore the Mann‐Whitney U test was used to assess differences in VAS confidence scores by correctness (as determined by NHTI recommendations).

## 3 RESULTS

### 3.1 Participant characteristics

Sociodemographic characteristics are shown in **Table** 1. A diverse sample of Nova Scotian adults participated in the study; mean (95% CI) age was 44 (41, 46) years and ranged from 19 to 95 years. The majority of participants identified as women (*n*=134, 60%), parents (*n*=158, 69%), and self‐identified as white (*n*= 164, 73%). There were significant differences in employment status (p=0.01) and whether or not a participant had been breastfed themselves as an infant (p<0.001) by gender, and significantly more women identified as parents (*p*=0.04).

**Table 1:** Sociodemographic characteristics of study participants.

**Characteristic**	***N*** †	**All** **n (%)**	**Women (*n*=134)** ‡ **n (%)**	**Men (*n*=90)** ‡ **n (%)**	***p*** §
Age, *years*	226				
*19‐29*		55 (24%)	27 (21%)	28 (31%)	0.331
*30‐44*		74 (33%)	44 (33%)	28 (31%)	
*45‐64*		66 (29%)	41 (31%)	23 (26%)	
*≥65*		31 (14%)	20 (15%)	11 (12%)	
Marital Status	227				
*Married or common‐law*		148 (65%)	92 (69%)	55 (61%)	0.291
*Single*		52 (23%)	25 (19%)	26 (29%)	
*Separated or divorced*		16 (7%)	8 (6%)	6 (7%)	
*Widow*		11 (5%)	8 (6%)	3 (3%)	
Annual household income before tax, *CAD$*	213				
*<$20,000*		44 (20%)	23 (19%)	18 (21%)	0.291
*$20,000‐$49,999*		46 (22%)	22 (18%)	24 (28%)	
*$50,000 ‐ $99,999*		70 (33%)	44 (36%)	25 (29%)	
*≥$100,000*		53 (25%)	34 (27%)	19 (22%)	
Self‐identified racial or cultural group	225				
*White*		164 (73%)	96 (73%)	63 (70%)	0.640
*Black*		34 (15%)	21 (16%)	13 (15%)	
*Chinese*		6 (3%)	1 (1%)	5 (6%)	
*Metis*		6 (3%)	3 (2%)	3 (3%)	
*First Nations*		5 (2%)	3 (2%)	2 (2%)	
*Arab*		4 (2%)	2 (2%)	2 (2%)	
*South Asian*		3 (1%)	2 (2%)	1 (1%)	
*Other* ¶		3 (1%)	2 (2%)	1 (1%)	
Born in Canada *(Yes)*	227	175 (77%)	106 (79%)	65 (73%)	0.294
Education	225				
*Some high school*		15 (7%)	8 (6%)	7 (8%)	0.714
*High school diploma*		35 (15%)	21 (16%)	14 (16%)	
*Some college or university*		51 (23%)	26 (20%)	22 (25%)	
*College diploma*		44 (20%)	26 (20%)	18 (20%)	
*Undergraduate degree*		41 (18%)	24 (18%)	16 (18%)	
*Some or completed post graduate*		39 (17%)	27 (20%)	11 (13%)	
Employment	227				
*Student*		27 (12%)	13 (10%)	14 (16%)	0.012
*Unemployed*		29 (13%)	15 (11%)	11 (13%)	
*Employed part time*		20 (9%)	18 (13%)	2 (2%)	
*Employed full time*		111 (49%)	60 (45%)	50 (56%)	
*Retired*		40 (17%)	28 (21%)	11 (13%)	
Parent *(Yes)*	228	158 (69%)	100 (75%)	55 (62%)	0.042
*Their child was breastfed (Yes)*	*158*	107 (69%)	73 (73%)	34 (63%)	0.197
*Child born ≥ 2012*	*158*	51 (22%)	35 (26%)	15 (17%)	0.096
Participant was breastfed themselves as an infant	227				
*Yes*		108 (48%)	57 (43%)	49 (55%)	<0.001
*No*		84 (37%)	64 (48%)	18 (20%)	
*Do not know*		35 (15%)	12 (9%)	22 (25%)	

† *N* differs due to participants skipping some questions.

‡ Total number of participants includes *n*=5 participants who were not categorized here as a man or woman: transfeminine (*n* =1), gender fluid (*n* =1), prefer not to say (*n* =2), and question skipped (*n* =1).

§ *x* ^2^ test, by gender, *p*<0.05.

¶ *Other* racial or cultural groups include Korean (*n*=1), Portuguese (*n* =1), Pacific Asian (*n* =1).

### 3.2 Infant and young child feeding knowledge

IYCF knowledge responses are shown in **Table** 2. The majority of participants stated that breastmilk was the best food for a new infant (*n*=192, 86%). The next most common response (9%) equated breastmilk and formula as the best food. The most common response for first solid food was vegetables (*n*=79, 36%), and only 7 participants referred to iron content in their reasoning for choosing a first solid food. The majority of participants (*n*=163, 72%) felt that infants and young children could consume cow's milk, with an average age of introduction of 13.7 (12.2, 15.2) months (NHTI recommendation is between 9‐12 months; Critch, 2014). Of those that felt cow's milk was appropriate, the majority (*n*=100, 62%) indicated that 3.25% milk fat was best. For foods to avoid before 24 months, the most common responses referred to choking hazards (23%), while 19% of participants could not list a contraindicated complementary food.

**Table 2:** Participant responses to infant and young child feeding knowledge questions.

**Infant or young child feeding topic**	***N*** †	***n* (%) or mean (95% CI)**
Best food for a newborn infant	223	
*Breastmilk*		192 (86%)
*Breastmilk or infant formula*		20 (9%)
*Infant formula*		2 (1%)
*Other*		9 (4%)
Exceptions to exclusive breastfeeding	229	
*Nothing*		142 (62%)
*Vitamin D drops*		24 (10%)
*Medication (if needed)*		1 (1%)
*Other* ‡		62 (27%)
Reasons not to breastfeed *(Yes)*	229	124 (54%)
Maternal reasons§	169	
*Illness and/or medication*		57 (34%)
*Breastfeeding difficulties/mother's choice*		34 (20%)
*Insufficient breast milk*		27 (16%)
*Drugs/alcohol*		22 (13%)
*HIV/AIDS*		6 (3%)
*Other* ¶		23 (14%)
Infant Reasons§	66	
*Illness*		21 (32%)
*Rejection of breast milk/allergies*		19 (29%)
*Latch issues*		18 (27%)
*Other* ††		8 (12%)
Age to terminate any breastfeeding*, months*	211	18.3 (16.7, 19.9)
Age to start complementary feeding, *months*	223	9.2 (8.2, 10.2)
Best first solid food	219	
*Vegetables*		79 (36%)
*Baby cereal*		62 (28%)
*Fruits*		31 (14%)
*Commercial baby food*		28 (13%)
*Meat and Meat Alternatives*		7 (3%)
*Other* ‡‡		12 (6%)
Reasoning for best first solid food type	158	
*Generally nutritious*		48 (30%)
*Soft texture*		42 (27%)
*Easy digestion*		28 (18%)
*Iron content*		7 (4%)
*Other* §§		33 (21%)
Age to introduce lumpy complementary foods, *months*	220	12.5 (11.3, 13.6)
Age to introduce family foods, *months*	224	18.2 (16.5, 19.8)
cow's milk is appropriate for infants and young children *(Yes)*	226	163 (72%)
Type of cow's milk to introduce (milk fat percentage)	161	
*3.25%*		100 (62%)
*2%*		29 (18%)
*1%*		12 (8%)
*Skim*		15 (9%)
*Any*		5 (3%)
Age to introduce cow's milk, *months*	160	13.7 (12.2, 15.2)
Age to start drinking from an open cup, *months*	221	20.4 (18.8, 21.9)
Contraindicated complementary foods§	227	
*Choking hazards*		87 (23%)
*None*		70 (19%)
*Potential allergens (nuts, seafood etc.)*		54 (14%)
*Highly processed foods (sodium, fat, sugar)*		52 (14%)
*Meat*		47 (12%)
*Honey*		19 (5%)
*Other* ¶¶		49 (13%)

† *N* differs due to participants skipping some questions.

‡ *Other* exceptions to exclusive breastfeeding include general solid foods (n=16), water (n=20), baby cereal (n=10), infant formula (n=8), juice (n=5), commercial baby food (n=2), iron supplement (n=1).

§ *N* differs because multiple responses were accepted; % calculated out of total responses.

¶ *Other* maternal reasons to not breastfeed include physical breast or nipple issues (i.e. “nipple too small”, “inverted nipple”, etc.; n=6), smoking (n=5), breast cancer (n=5), poor maternal diet (n=4), child adopted (n=1), tattoos (n=1), hepatitis (n=1).

†† *Other* infant reasons to not breastfeed include disease passed through breast milk (n=4), reflux (n=2), premature birth (n=1), child adopted (n=1)

‡‡ *Other* best first solid foods include “fresh”(minimally processed) foods (n=2), rice (n=2), custard (n=1), Cheerios™ (n=1), yogurt (n=1), sunflower butter (n=1), noodles (n=1), cheese puffs (n=1), rice crackers (n=1), amala (n=1)

§§ *Other* reasoning for best first solid food include conventional/past experience (n=5), doctor recommended (n=4), minimally processed (n=4), manufactured for babies (n=4), easy to prepare (n=3), allergen free (n=2), protein source (n=2), potassium source (n=1), high fat (n=1), satiating (n=1), necessary (n=1), can supplement breast milk (n=1), easily mixed with breast milk (n=1), versatile food (n=1), variety of foods important (n=1), inexpensive (n=1)

¶¶ *Other* contraindicated complementary foods include general fruits/vegetables (n=24), spicy foods (n=7), bread (n=4), raw meat/fish (n=3), crackers (n=3), foods that “produce intestinal discomfort” (n=2), most solid foods (n=1), rice (n=1), eba (n=1), fermented foods (n=1), chickpeas (n=1), raisins (n=1)

There was large variation in knowledge related to the timing and ages of various feeding milestones. The ideal age (mean (95% CI)) to terminate any breastfeeding was 18.3 (16.7, 19.9) months, however answers ranged from 2 to 84 months, and 52% of participants indicated a response of 12 months or less (data not shown). Sixteen respondents did not give a numerical answer but rather provided text that indicated responsive feeding (e.g. “as long as there is milk”).

### 3.3 Accuracy in responses: comparison to Nutrition for Healthy Term Infants recommendations

As shown in **Table** 3 **,** IYCF knowledge was generally low among all participants, indicating low awareness of the NHTI recommendations. While the majority of participants correctly identified breast milk as the ideal first food (*n*=192, 86%), only 32% (n=68) correctly stated that breastfeeding duration is recommended for 24 months and beyond. Only 10% of all participants correctly identified exceptions to exclusive breastfeeding (vitamin D supplementation, and medication as needed). Significantly more women correctly identified ideal first solid foods, ages to start complementary feeding and family food consumption, lumpy textures, appropriate milk fat percentage of cow's milk (if introduced), and contraindicated complementary foods compared to men (p<0.05). Men, younger adults (age 19‐29 years), and participants with lower annual household incomes (<$50,000) tended to have less accurate IYCF knowledge (p<0.05). There was no significant difference in IYCF knowledge by education level.

**Table 3:** Correct participant responses to infant and young child feeding knowledge questions, as determined by Nutrition for Healthy Term Infants recommendations.†

	**All** ‡ ***N* (%)**	**Gender** ***n* (%)**		**Age, *in years*** ***n* (%)**				**Household Income, *in thousands of CAD$*** ***n* (%)**				**Parental Status** ***n* (%)**		**Education** ***n* (%)**		
**Infant and young child feeding knowledge question** **(*Correct Recommendation)***		**Women**	**Men**	**19‐29**	**30‐44**	**45‐64**	**65+**	**<$20**	**$20‐<$50**	**$50‐<$100**	**≥$100**	**Parent**	**Non‐Parent**	**High_school** **or less**	**College or Undergraduate**	**Post Graduate**
Best food for a newborn infant *(Breastmilk)*	192 (86)	113 (85)	76 (88)	43 (78)^a^	61 (82)^ab^	58 (91)^ab^	28 (100)^b^	30 (70)^a^	41(89)^b^	63 (91)^b^	45 (87)^b^	134 (88)	58 (83)	41 (84)	112 (86)	36 (92)
Exceptions to exclusive breastfeeding *(Vitamin D drops; medication if needed)*	25 (10)	18 (13)	6 (7)	2 (4)	12 (16)	8 (12)	3 (10)	3 (7)	3 (7)	11 (16)	7 (13)	21 (13)	4 (6)	6 (12)	15 (11)	4 (10)
Age to terminate any breastfeeding *(≥24 months)*	68 (32)	37 (30)	28 (33)	17 (34)^ab^	30 (44)^a^	17 (27) ^ab^	4 (14)^b^	15 (36)	18 (40)	24 (39)	11 (23)	46 (33)	22 (31)	15 (32)	39 (31)	14 (40)
Age to start complementary feeding *(6 months)*	92 (41)	69 (52)^a^	22 (26)^b^	17 (32)	32 (44)	32 (49)	10 (35)	15 (35)	24 (52)	26 (39)	24 (45)	74 (48)^a^	18 (26)^b^	19 (40)	54 (41)	18 (46)
Best first solid food *(Iron fortified cereal; meat and meat alternatives)*	69 (37)	51 (45)^a^	18 (25)^b^	14 (32)	20 (32)	23 (41)	10 (42)	1 (29)	18 (50)	20 (35)	15 (32)	54 (40)	15 (28)	17 (38)	39 (35)	12 (41)
Age to start lumpy textures *(6‐9 months)*	82 (37)	68 (52)^a^	13 (15)^b^	14 (26)	33 (45)	27 (43)	7 (24)	13 (30)	17 (40)	25 (37)	25 (48)	70 (46)^a^	12 (18)^b^	18 (38)	46 (35)	17 (45)
Age to start family meal foods *(6‐12 months)*	119 (53)	89 (67)^a^	29 (33)^b^	17 (31)^a^	48 (66)^b^	38 (60)^b^	14 (45)^ab^	19 (43)^a^	19 (43)^a^	33 (48)^a^	41 (77)^b^	99 (64)^a^	20 (29)^b^	28 (58)	66 (50)	23 (59)
Age to introduce cow's milk *(≥ 9 months)*	122 (76)	73 (78)	47 (73)	30 (91)	41 (76)	34 (74)	16 (64)	23 (66)^a^	21 (66)^a^	35 (83)^ab^	36 (90)^b^	84 (74)	38 (81)	25 (66)	74 (82)	20 (69)
Types of cow's milk to introduce *(3.25% milk fat)*	100 (62)	67 (72)^a^	33 (50)^b^	16 (47)^a^	42 (76)^b^	29 (63)^ab^	12 (50) ^ab^	19 (56)	20 (41)	27 (63)	28 (70)	81 (71)^a^	19 (40)^b^	19 (49)	59 (66)	21 (72)
Contraindicated complementary foods *(*C*hoking hazards; honey; raw or undercooked meat, seafood or egg; unpasteurized milk or juice)*	139 (37)	96 (43)^a^	40 (28)^b^	36 (40)^a^	55 (44)^a^	38 (36)^a^	9 (16)^b^	36 (38)^a^	19 (28)^b^	42 (37)^a^	34 (44)^a^	97 (38)	42 (35)	27 (31)	96 (42)	14 (26)

† Within each comparison category (gender, age, household income, parental status, and education), labeled values in a row with a different letter differ, *p*<0.05 (*x* ^2^ test with Bonferroni post‐hoc test); absence of superscript letter indicates no significant difference.

‡ *N* differs due to participants skipping some questions.

### 3.4 Confidence in responses to IYCF knowledge questions

Participants' confidence in their answers to the IYCF knowledge questions, classified as *correct* or *incorrect,* can be found in **Table** 4. Mean (95% CI) confidence (out of 10) was generally high for all questions, 7.2 (6.9, 7.5), with no significant differences between correct and incorrect responses for the best food for a new infant, age to terminate any breastfeeding, and age to start family meal foods.

**Table 4:** Visual Analog Scale scores for self‐rated participant confidence in correct and incorrect responses to Nutrition for Healthy Term Infants recommendations.

**Infant or young child feeding topic**	***N*** †	**All** mean (95% CI**)**	**n**	**Correct** ‡	**n**	**Incorrect** ‡	***p*** §
Best food for a newborn infant	227	7.8 (7.5, 8.2)	191	8.1 (7.8, 8.4)	31	6.7 (5.4, 8.0)	0.148
Exceptions to exclusive breastfeeding	227	7.3 (7.0, 7.7)	25	8.3 (7.3, 9.2)	202	7.2 (6.8, 7.6)	0.032*
Age to terminate any breastfeeding	225	7.2 (6.8, 7.6)	67	7.5 (6.9, 8.2)	141	7.0 (6.5, 7.5)	0.089
Age to start complementary feeding	226	7.3 (6.9, 7.7)	92	8.1 (7.6, 8.6)	128	6.7 (6.2, 7.2)	0.001*
Best first solid food	225	7.2 (6.8, 7.5)	69	8.1 (7.6, 8.6)	116	6.9 (6.4, 7.4)	0.009*
Age to start lumpy textures	227	6.9 (6.6, 7.3)	82	7.5 (6.8, 8.0)	138	6.7 (6.2, 7.2)	0.034*
Age to start family foods	227	7.1 (6.7, 7.4)	119	7.3 (6.8, 7.8)	104	6.8 (6.3, 7.4)	0.305
Offering cow's milk to infants and young children¶	226	7.2 (6.8, 7.6)	‐	‐	‐	‐	‐
Contraindicated complementary foods (first food listed)	225	6.5 (6.0, 6.9)	72	7.2 (6.6, 7.9)	152	6.1 (5.6, 6.7)	0.041*

† *N* differs due to participants skipping some questions.

‡ accuracy of responses determined by Nutrition for Healthy Term Infants recommendations

§ Mann‐Whitney U test, accuracy of response determined using Nutrition for Healthy Term Infants recommendations.

¶ Nutrition for Healthy Term Infants document does not make explicit recommendations regarding the introduction of cow's milk; question not assessed for correctness.

* *p*<0.05

## 4 DISCUSSION

The aim of this study was to explore IYCF knowledge and confidence among a diverse sample of adult Nova Scotians because compliance with IYCF recommendations are low in Nova Scotia (Brown et al., 2013; Statistics Canada, 2018), and an unsupportive culture around breastfeeding has been identified as a barrier to improving feeding outcomes (Kirk et al., 2012). The results of this study revealed generally low knowledge of the content of the NHTI recommendations, with significantly lower knowledge among men, non‐parents, young adults (19‐29 years), and low‐income adults (<$50,000). Participants reported high confidence in their responses to knowledge questions regardless of accuracy in their responses.

### 4.1 IYCF knowledge deficits

The majority of participants provided incorrect answers regarding the exceptions to exclusive breastfeeding, age to terminate any breastfeeding, age to start complementary feeding, and both optimal and contraindicated complementary foods. For instance, the mean age to terminate any breastfeeding was 18.3 months, which is in agreement with previous research suggesting that people tend to underestimate the recommended total time for any breastfeeding (Fairbrother & Stanger‐Ross, 2010; Rempel & Rempel, 2004). Although not explored explicitly in this study, social disapproval of breastfeeding older infants and toddlers has been well documented (Cockerham‐Colas, Geer, Benker, & Joseph, 2012; Dowling & Brown, 2013; Newman & Williamson, 2018; Rempel, 2004; Stearns, 2011). Low awareness and potentially low acceptance of the recommendation to continue breastfeeding for toddlers may be contributing to reduced adherence to the recommendation for continued breastfeeding for 2 years and beyond.

Research on IYCF knowledge has focused mainly on breastfeeding, while complementary feeding remains comparatively under‐researched. This is important given that the majority of answers related to complementary feeding in this study did not match NHTI recommendations. Complementary feeding should begin at 6 months of age (Critch, 2014), however participants tended to over‐estimate the age to start complementary foods, with a mean (95% CI) age of 9.2 (8.2, 10.2) months, and nearly 60% of participants responded incorrectly (either above or below 6 months). Late introduction of complementary foods may increase the risk of nutritional deficiencies such as iron and zinc (Gibson, Ferguson, & Lehrfeld, 1998), and has been associated with increased feeding difficulties later in life, including decreased consumption of fruits and vegetables (Fewtrell et al., 2017).

The NHTI principles recommend iron‐rich foods, such as iron‐fortified baby cereal or meat and meat alternatives, as optimal first complementary foods, however the most common response for optimal first foods were vegetables (36%). While baby cereal was the second most commonly indicated food (28%), meat and meat alternatives comprised only 3% of responses. Contrary to NHTI recommendations, meat was listed as a contraindicated food before two years by 12% (*n=*47) of participants. It is unclear whether the general public understands that baby cereal is an acceptable first food because it has been fortified with important nutrients such as iron and zinc, as only 4% (*n*=7) of participants referred to iron content as rationale for cereal as an optimal first food. Participants generally over‐estimated the ages to introduce lumpy foods and family foods, and drastically over‐estimated the age to drink from an open cup (20.4 (18.8, 21.9) months) compared to the recommendation of 6 months or older (Critch, 2014), which again suggests a low awareness of NHTI recommendations for complementary feeding.

One surprising finding was that there was no relationship between educational attainment and IYCF knowledge, which may be indicative of the lack of infant feeding information available through public education in Canada. Most Nova Scotians will become parents or will care for a child in their lifetime: in 2011 only 15% of women in Canada aged 50 years or older had not had a biological child (not including adoption, step‐parenthood or surrogacy) (Provencher, Milan, Hallman, & D'Aoust, 2018). Yet dissemination of the NHTI principles and recommendations in Nova Scotia is reserved almost solely to caregivers during the pre‐ and postnatal period: through an online prenatal program, and postnatally through the *Loving Care* book series (birth to three years) (Nova Scotia Department of Health and Wellness, 2018). There are no standardized efforts to promote IYCF information among population segments outside of this perinatal period. One study found that only 20% of Nova Scotian high school students reported receiving some education around breastfeeding (Walsh, Moseley, & Jackson, 2008) and it is not known if students receive any education related to complementary feeding. This may be a missed opportunity given the various social influences on infant feeding decisions, which include family members (Mueffelmann et al., 2015), health care providers (Kornides & Kitsantas, 2013), and media (Hausman, 2007). Increased exposure to infant feeding recommendations through public education may help to improve infant feeding knowledge and attitudes in the general population. A recent systematic review of school‐based breastfeeding promotion interventions found that even short interventions (such as one to three sessions) may have the potential to improve perceptions and attitudes towards breastfeeding, as well as intention to breastfeed in the future (Glaser, Roberts, Grosskopf, & Basch, 2016). This lack of exposure is particularly relevant for young men (aged 19‐29 years), who were found to have the lowest IYCF knowledge, since male partners have been shown to be key influencers for infant feeding decisions (Rempel & Rempel, 2004; Vaaler et al., 2011). The cause of this knowledge gap is complex; Brown and Davies found that male partners often felt excluded by health care professionals from breastfeeding education and promotion, despite their interest in the information and desire to support their partners (Brown & Davies, 2014).

### 4.2 Confidence in IYCF knowledge

The VAS scores for confidence in responses to knowledge questions revealed that participants generally felt confident about their responses regardless of whether the response was correct. Family members have been shown to have substantial influence on infant feeding practices (Mueffelmann et al., 2015), and high confidence in incorrect responses could result in the perpetuation of non‐evidence based decisions and non‐compliance to NHTI recommendations. This is a novel finding, and in line with above, provides additional rationale to increase the availability and dissemination of IYCF recommendations to the general public through avenues such as public schools (Walsh et al., 2008) or large‐scale public health campaigns.

### 4.3 Consumption of cow's milk

The NHTI principles do not explicitly recommend the consumption of cow's milk, but rather provide guidance on the appropriate timing of introduction (between 9‐12 months) and type of milk (3.25% milk fat), if parents choose to provide cow's milk to their children. Dairy consumption, particularly of fluid milk, has been steadily decreasing for the past 20 years in Canada (Statistics Canada, 2017b). In our findings, 28% of study participants felt that cow's milk was not appropriate for infants and young children. Future research should examine why so many participants opposed young children's consumption of cow's milk, and whether this was related to perceptions of nutritional value, the manufacturing and processing of cow's milk (i.e. environmental concerns, animal welfare), or some other reason (McCarthy, Parker, Ameerally, Drake, & Drake, 2017).

### 4.4 Moralized infant feeding

‘Breast is best' is a mainstay in public health messaging, but seems to be receiving backlash from mothers and activists in recent years (Mullan, 2015). Infant feeding has increasingly become a moralized activity, with mothers identifying both perceptions of inadequate parenting associated with not breastfeeding, as well as feelings of humiliation and shame associated with breastfeeding (Thomson, Ebisch‐Burton, & Flacking, 2015). Nearly one in ten participants in our study indicated that human milk or infant formula were equally optimal as first foods for a newborn, which is in agreement with another similar study that explored this topic using closed‐ended questionnaires (Fairbrother & Stanger‐Ross, 2010), however, 95% of these participants in our study identified as women. Because women had greater IYCF knowledge than men in almost all other areas of NHTI guidelines, it is possible that this finding may be more suggestive of the controversial nature of infant feeding and resistance to the idea of moralized infant feeding, as opposed to a true knowledge gap (Lee, 2007). Results from a recent prospective questionnaire study indicate that personal or vicarious experience with breast milk substitutes were predictors of negative breastfeeding outcomes at 6 weeks postnatal (Bartle & Harvey, 2017). Nearly 30% of parents in our study reported that their baby was not breastfed, which may have introduced personal bias into their responses. However, reasoning for responses was not explored in this study and further research is needed to understand why more women equated human milk and infant formula in this population.

### 4.5 Study strengths and limitations

Convenience sampling has potential to introduce sampling bias, however we attempted to mitigate potential bias by purposively selecting diverse research collection sites. Another limitation is language comprehension: we did not screen for English comprehension as part of the recruitment process, therefore it is possible that some participants did not fully understand all questions. However, 99.7% of Nova Scotians have indicated knowledge of the English language, and 96.6% of households use English as a primary language (Statistics Canada, 2017a), therefore this limitation should not affect population group representation in our study. Strengths of this study include the size and diversity of the study sample, including in age, household income, immigration status, education level, urban and rural residency, cultural group, and parental status. Another strength is the exploration of an under‐researched topic, particularly the inclusion of infant feeding knowledge questions beyond breastfeeding.

## 5 CONCLUSIONS

Adherence to the Canadian IYCF guidelines, NHTI, is low despite the well‐documented benefits for optimal infant growth and development (Fegan et al., 2016). In this study, IYCF knowledge deficits were greater among men, young adults (19‐29 years), non‐parents, and low‐income groups, particularly in regards to age to start, and the best and worst foods for complementary feeding. Surprisingly there was no difference in knowledge or confidence in responses by education level. Regardless of alignment with NHTI principles, most participants felt confident about their responses. Low awareness of the NHTI principles among the general public may contribute to low acceptance of, and adherence to, these recommendations. Increasing availability and dissemination of IYCF recommendations, not only to new parents, but to the wider population, may help to create a more supportive culture around optimal infant feeding in Nova Scotia and beyond.

## CONFLICTS OF INTEREST

KC and KCW declare no conflicts of interest. The funder had no role in the study design, data collection and analysis, decision to publish, or preparation of the manuscript.

## ETHICAL STATEMENT

This study was approved by the Mount Saint Vincent University Research Ethics Board (MSVU UREB 2017‐068).

## CONTRIBUTIONS

KCW conceived the study and obtained funding, aided in data analysis and interpretation, and is responsible for the final version of the manuscript. KC collected, analyzed, and interpreted data, and drafted first version of this manuscript. Both authors reviewed manuscript revisions and contributed to the intellectual content of the manuscript.
